# Revolutionizing the Role of Solar Light Responsive BiVO_4_/BiOBr Heterojunction Photocatalyst for the Photocatalytic Deterioration of Tetracycline and Photoelectrocatalytic Water Splitting

**DOI:** 10.3390/ma16165661

**Published:** 2023-08-17

**Authors:** Shelly Singla, Pooja Devi, Soumen Basu

**Affiliations:** 1 Materials Science and Sensor Application, Central Scientific Instruments Organisation, Chandigarh 160030, India; 2 School of Chemistry and Biochemistry, Thapar Institute of Engineering and Technology, Patiala 147004, India

**Keywords:** BiVO_4_/BiOBr, photocatalytic decomposition, tetracycline, solar light, photoelectrocatalytic water splitting

## Abstract

In this study, a series of BiVO_4_/BiOBr composites with varying mole ratios were successfully synthesized using a hydrothermal method. The in-situ synthesis strategy facilitated the formation of a close interfacial contact between BiVO_4_ and BiOBr at the depletion zone, resulting in improved charge segregation, migration, reduced charge recombination, enhanced solar light absorption capacity, promoting narrow band gap, and large surface area. This study investigates the influence of different mole ratios of BiVO_4_ and BiOBr in a BiVO_4_/BiOBr nanocomposite on the photocatalytic degradation of tetracycline (TC), a pharmaceutical pollutant, and photoelectrocatalytic water splitting (PEC) under solar light irradiation. Maximum decomposition efficiency of ~90.4% (with a rate constant of 0.0159 min^−1^) for TC was achieved with 0.5 g/L of 3:1 BiVO_4_: BiOBr (31BVBI) photocatalyst within 140 min. The degraded compounds resulting from the TC abatement were analyzed using GC-MS. Furthermore, TC standards exhibited 78.2% and 87.7% removal of chemical oxygen demand (COD) and total organic carbon (TOC), respectively, while TC tablets showed 64.6% COD removal and 73.8% TOC removal. The PEC water splitting experiments demonstrated that the 31BVBI photoanode achieved the highest photocurrent density of approximately 0.2198 mA/cm^2^ at 1.23 V vs. RHE, resulting in the generation of approximately 1.864 mmolcm^−2^ s^−1^ of hydrogen, while remaining stable for 21,600 s. The stability of the photocatalyst was confirmed by post-degradation characterizations, which revealed intact crystalline planes, shape, and surface area. Comparisons with existing physicochemical methods used in industries indicate that the reported photocatalyst possesses strong surface catalytic properties and has the potential for application in industrial wastewater treatment and hydrogen generation, offering an advantageous alternative to costly and time-consuming processes.

## 1. Introduction

Environmental and energy challenges are gaining prominence on a global scale owing to a rapid expansion of the social economy and the effects of human activity [1,2]. Due to escalating industrialization and unnecessary use of human resources, water pollution has recently emerged as one of the most significant global environmental issues [3,4]. Tetracycline (TC) has been widely used as a broad-spectrum antibiotic medication in human medicine, animal farming, and agriculture to prevent and cure diseases triggered by bacterial infections [5]. However, TC residues present in the water offer major harm to humans, animals, plants, and microorganisms and also disrupt the ecological balance [6]. People often consume water laced with TC, which can alter the composition of their gut flora and greatly increase the risk of developing noxious diseases including fatty liver, metabolic syndrome, autism, and other neurological disorders [7].

TC cannot be completely removed by conventional water treatment methods such as membrane filtration, adsorption, microwave catalysis, electro-Fenton process [8], etc due to limited biodegradability, stable structure, and higher toxicity [9]. Moreover, these methods lead to the production of secondary pollutants at the last stage of treatment [10]. Hence, it is necessary to find out an effective method to eliminate TC from the water completely. In this context, photocatalysis based on the direct exploitation of solar energy for the total mineralization of refractory organic pollutants into carbon dioxide and water in an aquatic environment is regarded as a promising technique. Moreover, it is safe, non-toxic, energy-saving, and very effective [11,12].

Apart from environmental pollution, the energy crisis issues brought on by the excessive use of fossil fuels is also one of the most significant obstacles preventing human society from expanding [13]. The burning of fossil fuels releases hazardous gases into the environment, such as ammonia, carbon dioxide, methane, etc [14]. Many scientific experts are concentrating on discovering ecologically suitable and renewable energy sources to replace fossil fuels [15]. Since hydrogen is a clean, affordable, and durable energy source, it is frequently viewed as the most suitable alternative energy carrier [16]. Photoelectrocatalytic (PEC) water splitting has been proposed as a viable and one of the most efficacious methods to evolve hydrogen due to its tendency to utilize abundant water resources and solar light, and the fact that the process is free of secondary pollution [17].

However, the efficient photocatalytic degradation of organic pollutants and PEC water splitting requires the suitable design of visible light-responsive photocatalysts [18]. Rare earth metals, a group of elements comprising lanthanides and scandium, also offer a host of invaluable advantages across various sectors. Their exceptional magnetic properties drive the production of compact, high-performance magnets vital for electric vehicles, wind turbines, and efficient motors [19]. These metals also enhance the efficiency of lighting and display technologies through phosphors, enabling energy-saving LED screens and vibrant color displays. In the realm of electronics, rare earth metals are instrumental in miniaturization and improved performance of devices such as smartphones and laptops. Moreover, their role in catalysis supports environmentally friendly processes in industries such as petroleum refining and pollution control [20]. In essence, rare earth metals stand as indispensable components, fostering technological innovation, sustainable energy solutions, and overall global progress. Numerous inorganic semiconductors have also been explored and exploited in the photocatalytic degradation of organic pollutants and photoelectrocatalytic water splitting [21,22]. Amongst these, BiVO_4_ has generated significant attention owing to its chemical stability, easier preparation, low cost, and environmental friendliness [23]. In addition, it has visible light absorption capacity and possesses a narrow band gap. However, its practical applicability is constrained by the poor charge transfer rate and quick recombination of photogenerated charge carriers [24]. In order to ameliorate these issues, BiVO_4_ should be hybridized with another semiconductor photocatalyst as the heterojunction formation increases the lifetime of the charges owing to the synergistic effect [25]. Utilizing this approach, about 74.0%, and 86.7% of degradation of fipronil, and ciprofloxacin were observed with BiVO_4_/g-C_3_N_4_/Ag, CuS/BiVO_4_, respectively, under visible light irradiation within 120 min, 90 min, respectively [10,26]. Likewise, for PEC water splitting, BiVO_4_/WO_3_ photoanode is reported to exhibit a photocurrent density of 0.002 mA/cm^2^ at 1.23 V vs. Ag/AgCl [23]. BiOBr is a typical p-type semiconductor photocatalyst and is the catalyst of choice for heterostructure due to its excellent stability, environmental friendliness, non-toxicity, and appropriate band gap. It has a unique lamellar structure with [Bi_2_O_2_]^2+^ sheets interspersed with two-layer bromine atoms (tetragonal matlockite structure) [27]. This shape offers enough room to polarize the associated kernel and orbital, making it easier to separate photo-produced carriers for effective photocatalytic/PEC activity [10,28,29]. Furthermore, the visible light activity of other semiconductors can be improved by coupling with BiOBr. WS_2_/BiOBr, CuBi_2_O_4_/Bi/BiOBr exhibited 85% of degradation of metronidazole, and 81% for ciprofloxacin under visible light [28,29].

Although, many photocatalytic remediation experiments using pure BiVO_4_, BiOBr, and BiVO_4_/BiOBr couple and PEC experiments with BiVO_4_, BiOBr and their hybridization with the other semiconductors have been published in the literature. However, the impact of different mole ratios of BiVO_4_ and BiOBr has not been explored for the photocatalytic abatement of the noxious pollutant, TC under natural sunlight and PEC water splitting under solar light simultaneously. Moreover, the degradation efficiency of the BiVO_4_/BiOBr photocatalyst has not been compared under different light sources and with the commercial TiO_2_-P25 photocatalyst. The degraded products formed after the photocatalytic degradation of TC have not been studied yet.

Herein, this study is focused on synthesizing different mole ratios of the BiVO_4_/BiOBr couple via the hydrothermal method. Thereafter the activity of the synthesized photocatalyst has been tested for the photocatalytic abatement TC under natural sunlight as well as for PEC water splitting. The impact of different mole ratios on the photocatalytic degradation of the organic pollutant, TC, and PEC water splitting under solar light have been explored. Numerous experiments such as the impact of photocatalyst concentration, scavenger studies, kinetic studies, the impact of pH, Mott–Schottky, linear sweep voltammetry (LSV), reusability, chronoamperometry, and stability studies have been carried out. Mineralization studies with commercial TC and real TC tablets have also been executed to analyze the efficacy of the fabricated photocatalyst. Degraded products have been scrutinized by gas chromatography-mass spectrometry (GC-MS). Additionally, based on all the experiments, the catalytic mechanism has been proposed.

## 2. Materials and Methods

Sodium hydroxide pellets (NaOH) were purchased from Merck. Bismuth nitrate pentahydrate (Bi(NO_3_)_3_.5H_2_O), ethanol, nitric acid (HNO_3_), and acetic acid were bought from Spectrochem. From Loba Chemie, potassium bromide (KBr) was procured. The standard TC was bought from Sigma Aldrich, while the real TC tablet (company-Abbott, Tetracycline capsules I.P. Restecline 250) with a concentration of 250 mg was purchased from a local medical store. Deionized water (DI water) (18 MΩcm^−1^) was employed to prepare all the solutions. Analytical-grade chemicals were used in an unadulterated way and without any further refinement.

### 2.1. Synthesis of BiVO_4_

The hydrothermal route was utilized to synthesize BiVO_4_. Initially, 5 mmol of Bi(NO_3_)_3_.5H_2_O was dissolved in 10 mL of 4 M HNO_3_ under continuous stirring. Subsequently, 5 mmol of NH_4_VO_3_ was dissolved in 10 mL of 2 M NaOH in a separate flask. Then, 0.25 g of C_18_H_29_NaO_3_S was added to each of the prepared solutions. Following that, both solutions were mixed together and the pH of the resulting solution was maintained to neutral with NaOH. The resultant suspension was heated in a Teflon-lined autoclave for 1.5 h at 200 °C. A yellowish-colored powder was obtained, centrifuged, washed with DI water, and dried in an oven at 70 °C. The finally attained sample was termed BV.

### 2.2. Fabrication of BiVO_4_/BiOBr Hybrid

Hydrothermal synthesis was followed to fabricate a BiVO_4_/BiOBr (BVBI) couple with various mole ratios. Initially, 0.005 mol of Bi(NO_3_)_3_.5H_2_O was dissolved into 7.5 mL of acetic acid. In another flask, 0.005 mol of KBr was dissolved into 75 mL of DI water under vigorous stirring. Then, both solutions were blended together and 0.005 mol of BiVO_4_ was dissolved into the resultant solution. Then the obtained solution was subjected to sonication for 30 min. The solution was heated at 120 °C for 5 h in a Teflon line autoclave, thereafter. The resultant suspension was dried at 70 °C in a hot air oven after 3–4 washes with DI water and ethanol. The obtained pale yellowish powder was named as 1:1BiVO_4_:BiOBr (11BVBI). Likewise, the number of chemicals required to synthesize BVBI with different molar ratios (1:3 and 3:1) were calculated and were designated as 13BVBI and 31BVBI.

For the comparison, white-colored bare BiOBr was prepared analogously without the addition of BiVO_4_ and was considered as BI [10].

### 2.3. Characterization Techniques

Section S1.1 (Supporting Information) details the characterization procedures.

### 2.4. Photocatalytic Remediation of TC

The refractory pharmaceutical pollutant, TC, was photocatalytically decomposed in the existence of natural sunlight to gauge the activity of the prepared photocatalysts. A 5 mg of the photocatalyst was dispersed into 10 mL of 10 ppm solution of TC. The mixture was magnetically agitated in the dark for 80 min prior to the irradiation to accomplish adsorption-desorption equilibrium between the pollutant and photocatalyst. Finally, the photocatalytic decomposition was initiated by illuminating the reaction mixture under sunlight for 140 min. The experiments were performed in September–October, and the LICOR Pyranometer determined that the sunlight intensity ranged between 700–760 W/m^2^ during this time. About 1.5 mL aliquots of the solutions were centrifuged to extract the photocatalyst at predetermined intervals. The experiments were repeated three times, and the graphs are plotted with error bars showing ~5% data source error. A UV-visible spectrophotometer was employed to record changes in the absorption band of TC at a characteristic wavelength (λ_max_ = 360 nm) in order to evaluate the filtrates and to determine the concentration of the pollutant. The decomposition activity can be assessed by Equation (1).

% Deg = {(Ab_0_ − Ab_t_)/Ab_0_} × 100 = {(*C*_0_ − *C_t_*)/*C*_0_} × 100 (1)

where, % Deg, C_0_, and Ab_0_, are decomposition proficiency, initial concentration, and absorbance, respectively. While, C_t_, and Ab_t_ are the concentration and absorbance at time t, respectively.

The effectiveness of the photocatalytic remediation of TC was also evaluated using chemical oxygen demand (COD) and total organic carbon (TOC) analyses. Before the analyses, a 200 mL solution of TC containing 100 mg of 31BVBI photocatalyst was illuminated under sunlight for 140 min. COD was gauged by the titration method, while the mineralization was estimated using TOC eradication. Equations (2) and (3) were used to compute the% COD and TOC elimination.

% COD = {(COD_i_ − COD_f_)/COD_i_} × 100 (2)

% TOC = {(TOC_i_ − OC_f_)/TOC_i_} × 100 (3)

Herein, COD_i_, COD_f_, TOC_i_, and TOC_f_, stand for the initial and final COD, and TOC, respectively.

### 2.5. PEC Studies

In order to conduct PEC investigations, the electrochemical workstation (AUTOLAB, Metrohm) was outfitted with a standard three-electrode configuration, and the solar simulator (LOT Quantum design) was utilized to test out PEC activity of the prepared electrodes under one sun illumination. Spray-coated BV, BI, 11BVBI, 13BVBI, and 31BVBI onto Indium Tin Oxide (ITO) were used as photoanodes (working electrodes), Pt wire (counter electrode), and Ag/AgCl (reference electrode). According to the photo-response behavior, the coating of the photocatalyst was optimized for 5, 10, and 15 layers. 0.5 M Na_2_SO_4_ solution (pH = 7) was utilized as a supporting electrolyte. The solar light intensity of 100 mW/cm^2^ was used for performing photo-response tests which included LSV, electric impedance spectroscopy (EIS), chronoamperometry, and transient photocurrent. In both dark and light environments, impedance spectra were recorded at open circuit potential (OCP) with an AC perturbation signal of 10 mV. Additionally, Mott–Schottky measurements were performed on the constructed electrodes in a frequency-potential scan mode of 100 kHz. All potentials were analyzed using the Ag/AgCl as a reference electrode, and their conversion to the RHE scale was completed using the Nernst equation. Lastly, the stability study was carried out for 21,600 s (6 h) to evaluate the stability of the fabricated electrode.

## 3. Results and Discussion

### 3.1. X-Ray Photoelectron Analysis (XPS)

**Figure 1:**
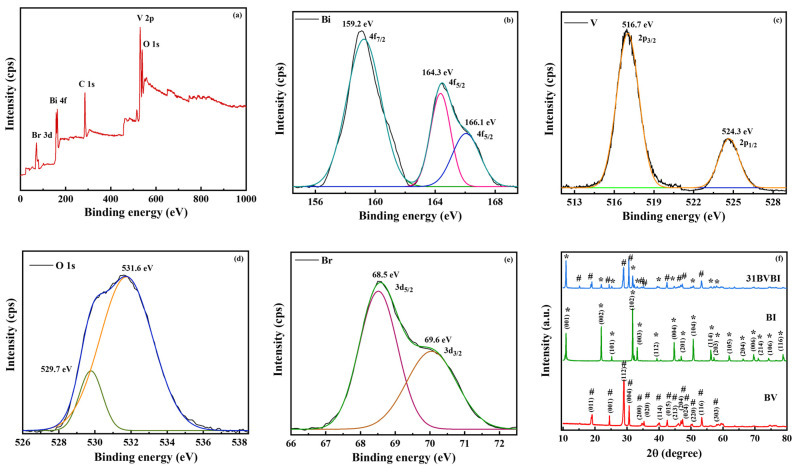
XPS spectra of 31BVBI nanocomposite, (**a**) the survey spectrum, (**b**) Bi 4f, (**c**) V 2p, (**d**) O 1s, (**e**) Br 3d, and (**f**) XRD plot of BV, BI, and 31BVBI photocatalyst where # and * denote peaks of BV, and BI, respectively.

The XPS analyses were performed to scrutinize the local electronic environment of the composite along with the binding energy, oxidation state, and chemical composition of the elements. Figure 1a representing the survey spectrum of 31BVBI nanocomposite specifies the existence of Bi, O, V, and Br at corresponding binding energies. A supplementary peak of the adventitious carbon at a binding energy of 284.5 eV was seen as all peaks were calibrated concerning the C 1s. Deconvolution was executed according to the least square Gaussian-fit model to fit the peaks. In the core level spectrum of Bi, and V, the existence of peaks at the binding energies of 159.2, 164.3, and 166.1 eV (Figure 1b), 516.7, and 524.3 eV (Figure 1c) correspond to Bi 4f_7/2_ and 4f_5/2_, V 2p_3/2_ and 2p_1/2_ attribute to +3, and +5 oxidation state of Bi, and V, respectively [30,31,32]. The peak at a binding energy of 166.1 eV of Bi is attributed to the Bi-O band of the 31BVBI photocatalyst [33]. O 1s XPS spectra consist of a dual peak with binding energy 529.7, and 531.6 eV, which is accredited to the Bi-O and Bi-O-Bi band of BVBI nanocomposite, respectively (Figure 1d) [33]. The dual peak of Br at binding energy 68.5 and 69.6 eV correspondent with energy states 3d_5/2_ and 3d_3/2_ were ascribed to a −1 oxidation state as depicted in Figure 1e [34,35]. Thus, the XPS spectrum manifested the successful fabrication of the hetero-composite.

### 3.2. X-Ray Diffraction (XRD) Analysis

The XRD was performed to validate the successful synthesis, crystallite phase, purity, and structural analysis of the fabricated photocatalysts [36]. Figure 1f represents the XRD pattern of BV, BI, and 31BVBI photocatalyst and the XRD of 11BVBI and 13BVBI composite is presented in Figure S1 (Supporting Information). The crystallinity of hexagonal BV was emphasized by the presence of sharp peaks indexed to 2θ values at 19.0°, 24.5°, 28.8°, 30.6°, 34.8°, 35.2°, 40.0°, 42.6°, 46.1°, 46.9°, 47.4°, 50.1°, 53.2°, and 58.3° attributing to the crystal planes (011), (102), (112), (004), (200), (020), (211), (015), (123), (204), (024), (220), (301), and (303), respectively (JCPDS card-83-1699). The efficient fabrication of tetragonal BI was confirmed by the presence of peaks indexed at crystal planes (001), (002), (101), (102), (003), (112), (004), (201), (104), (114), (203), (105), (204), (006), (214), (106), and (116) agrees to 2θ value of 10.9°, 21.9°, 25.1°, 31.7°, 33.1°, 39.3°, 44.6°, 47.6°, 50.6°, 56.1°, 58.0°, 61.8°, 66.2°, 69.5°, 71.0°, 74.1°, and 78.7°, respectively (JCPDS-01-078-0348). The existence of sharp peaks confirms the crystal nature of the fabricated photocatalysts. The incidence of both types of diffraction peaks certified the efficacious fabrication of the nanocomposite (BVBI) without the presence of any impurities.

### 3.3. Photoluminescence Study (PL)

The PL study was executed to investigate the separation, allocation, and reintegration rate of the photogenerated charges. A lower recombination rate, more carrier charge migration capacity, and effective charge separation are all necessary for an efficacious photocatalyst [37]. Lower PL signal intensity of photocatalysts results in less photoexcited carrier recombination and higher charge migration efficacy, which is beneficial for the PEC performance of the photocatalyst [38]. Figure S2 represents the PL spectra of the synthesized photocatalysts. The 31BVBI photocatalyst exhibited weaker PL emission intensity amongst the fabricated photocatalysts indicating a lower recombination rate while bare BI and BV exhibited the strongest PL signal. Due to the synergetic effect between both semiconductors, 31BVBI can significantly enhance the number of charge capture sites with the addition of BI, hence boosting the charge separation efficiency.

### 3.4. Surface Area Analyses

**Figure 2:**
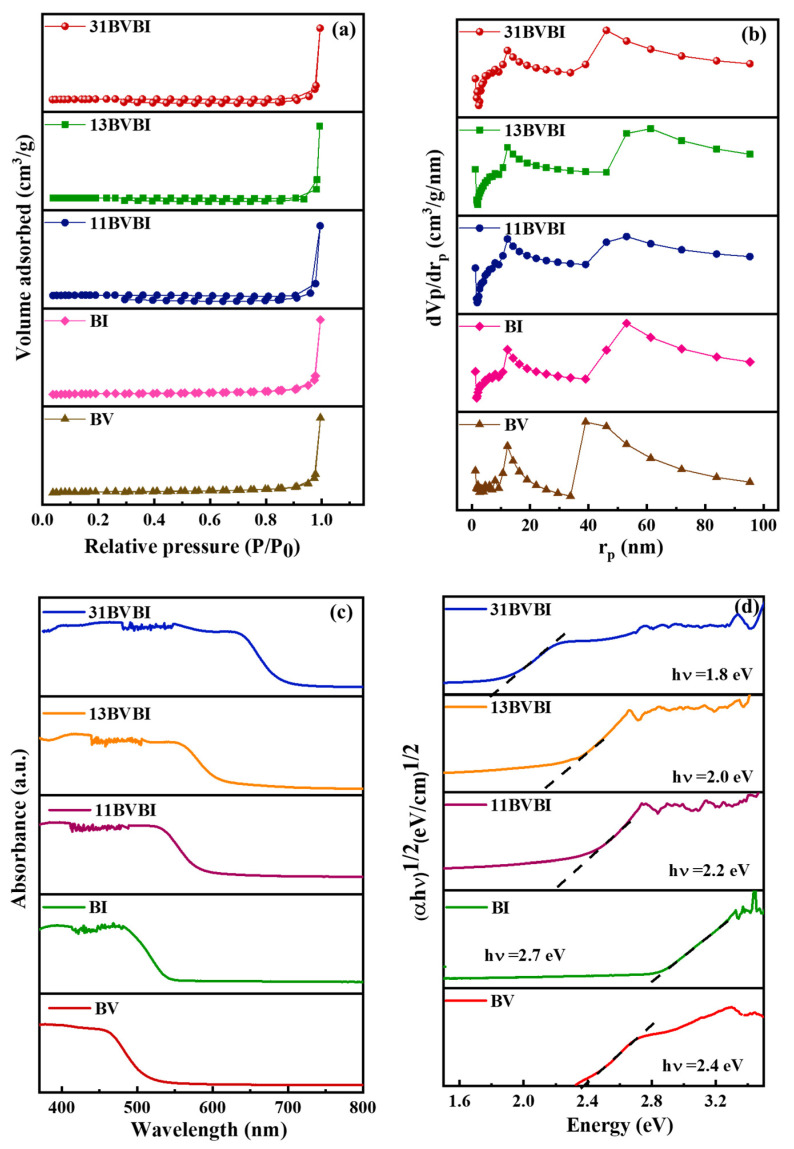
(**a**) Nitrogen sorption isotherms, (**b**) BJH plot, (**c**) absorbance spectrum, and (**d**) band gap energies of the as-prepared photocatalysts.

Surface area analyses are one of the important parameters for PEC/photocatalysis [39]. The concept of monolayer formation of the nitrogen gas molecules on a solid surface was employed to calculate the precise surface area. Barrett–Joyner–Halenda (BJH) method was executed to monitor the pore size distribution. The fabricated photocatalysts have a type-IV N_2_ adsorption curve together with an H3 hysteresis regression loop, signifying the mesoporous nature (Figure 2a,b). Mean pore diameter, pore volume, and surface area results are tabularized in Table 1. These results suggest that surface area was highest for 31BVBI nanocomposite owing to the formation of heterojunction amongst all the fabricated photocatalysts. Higher surface area facilitates a greater number of surface-active sites [40]. Furthermore, mesopores offer effective pathways for mass transport and improve the interfacial contact between the pollutant and the surface of the catalyst, therefore higher photocatalytic degradation activity [41]. Additionally, the H_2_ production rate also increases with the intensification in the surface area of the photocatalyst.

### 3.5. Absorption Studies

UV-Visible diffused reflectance spectroscopy (DRS) was performed to assess the optical absorption properties of the synthesized nanocomposites. On absorbing a photon of energy greater than the band gap of the photocatalyst, the excitation of the electrons occurs from its valence band to the conduction band [42]. The transition of the electron, therefore, results in the sharp rise in the absorbance tendency of the photocatalyst to the wavelength ascribing to the band gap energy. Figure 2c validates that nanocomposites possess better visible light absorption intensity than pristine BV and BI. Enhanced light absorption may be due to the synergistic effect of both semiconductors.

Kubelka–Munk equation (Equation (4)) was used to determine the band gap of the prepared photocatalysts.

(αhν)^1/2^ = hν − E_g_ (4)

where, h, α, ν, and E_g_ stand for Planck’s constant, absorption coefficient, the frequency of light, and intercepts of tangents of plots illustrating band gap.

The band gap can be assessed by extrapolating the linear area of the curve to the energy axis. E_g_ values for BV, BI, 11BVBI, 13BVBI, and 31BVBI were determined to be 2.4, 2.7, 2.2, 2.0, and 1.8 eV as represented in Figure 2d. These findings suggest that 31BVBI nanocomposite offers a narrow band gap and extensive visible light absorption capability among the synthesized catalysts. The electronic properties caused by a successful interface between both photocatalysts are responsible for the narrow band gap, which is essential for boosting PEC/photocatalytic activity.

### 3.6. Scanning Electron Microscopy (SEM)

**Figure 3:**
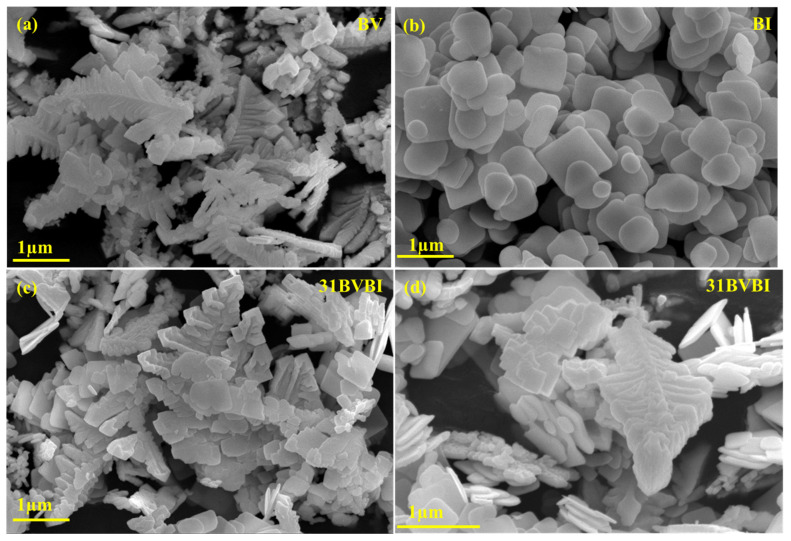
The SEM images of (**a**) BV, (**b**) BI, and (**c**,**d**) 31BVBI nanocomposite at different magnifications.

SEM was carried out to ascertain the surface morphology of the 31BVBI nanocomposite. The results revealed that BV possesses fern-petal-like morphology while BI has disc-shaped morphology (Figure 3a,b). As demonstrated in Figure 3c,d, 31BVBI photocatalyst possess discs decorated onto fern petals. The efficacious formation of the 31BVBI nanocomposite was thus confirmed by the existence of both types of structures. Additionally, these results show an excellent distribution of BI onto BV, which is advantageous for a heterojunction photocatalyst.

### 3.7. Energy-Dispersive X-ray Spectroscopy (EDS) and Elemental Mapping

**Figure 4:**
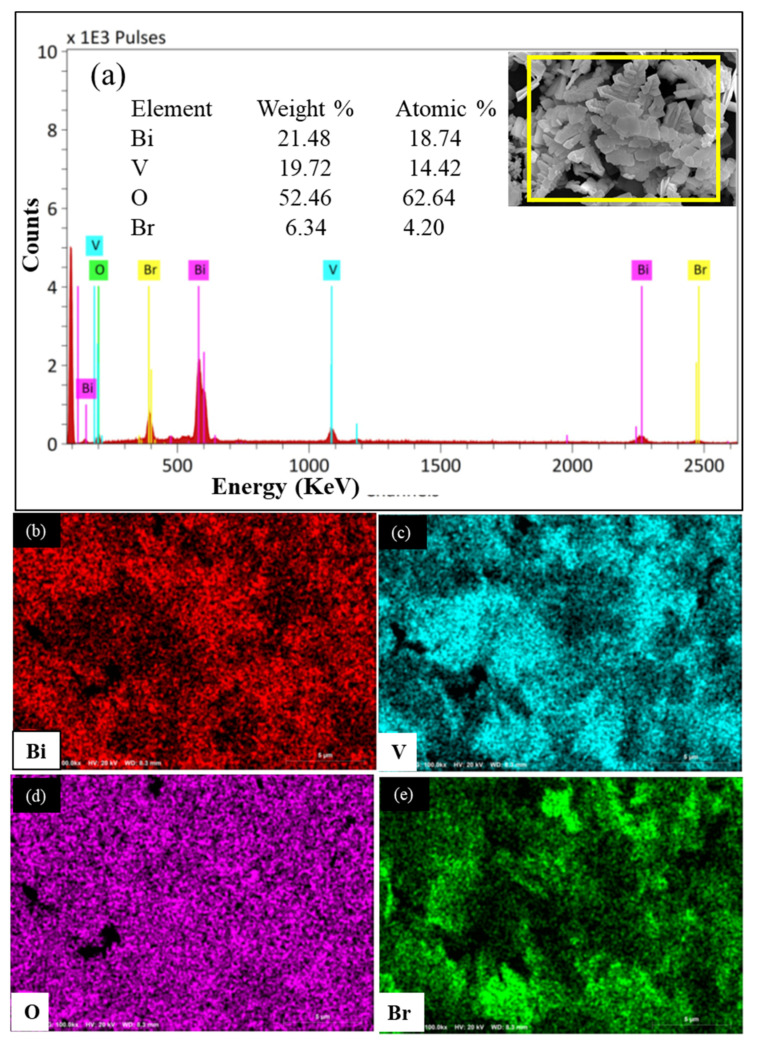
(**a**) EDS spectrum of 31BVBI with the corresponding SEM micrograph, and (**b**–**e**) elemental mapping.

EDS was executed to evaluate the elemental composition, distribution, and purity of the photocatalyst. The EDS spectra exhibit a strong signal of Bi, Br, V, and O, all of which were embedded inside the selected regions of 31BVBI photocatalyst as represented in Figure 4a. The absence of any additional elemental peak in the spectrum implies that there were no impurities on the surface of the synthesized composite, supporting the successful synthesis of the material [43]. Elemental mapping reveals that all the elements were uniformly dispersed and their distribution agrees well with the SEM image of the composite which is advantageous for a heterojunction nanocomposite (Figure 4b–e).

### 3.8. Photocatalytic Abatement Studies

**Figure 5:**
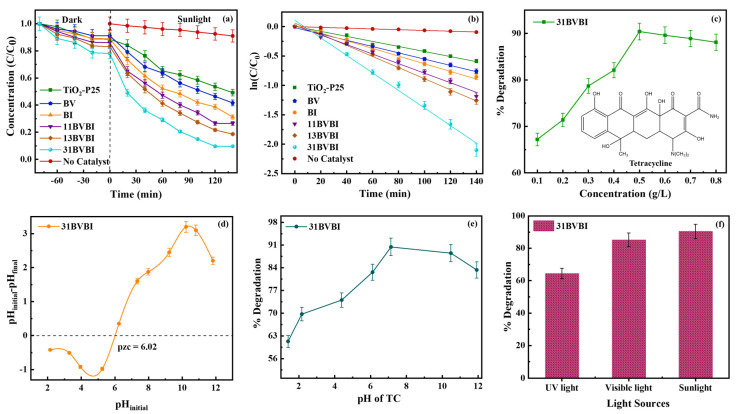
(**a**,**b**) The kinetic analyses of the different photocatalysts for the deterioration of the TC, (**c**) effect of the photocatalyst’s concentration on the removal of the TC pollutant, (**d**) pzc of the 31BVBI photocatalyst, (**e**) influence of pH on the degradation efficacy, and (**f**) impact of the different light sources.

The activity of the photocatalysts was gauged by the photocatalytic deterioration of TC under the illumination of sunlight for 140 min. Figure 5a,b depicts the photocatalytic abatement results of the detrimental TC. Little change in the concentration of TC was observed without the photocatalyst, indicating that self-degradation of the pollutant (9%) is less under sunlight owing to its stable nature. However, the highest decomposition of TC was observed to be 90.4% in the existence of 0.5 g/L of the 31BVBI photocatalyst. Two major factors attribute to the increased photocatalytic performance of the 31BVBI composite. Before the degradation through photocatalysis, TC should be thoroughly adsorbed on the surface of the nanocomposite. Higher adsorption of pollutants occurs due to electrostatic interaction between the negatively charged surface of BI (due to the Br-layer) and the positively charged surface of TC (due to the cationic moiety of TCH_3_^+^ and TCH_2_^+^). Additionally, the unique structure of BI promotes the segregation of the charge carriers in BV. However, the capacity of the holes and electrons to oxidize and reduce may be deprived owing to the excess amount of BI present in the composite, which might also prevent electron transport and push the conduction band of BV to a more positive position [44]. Therefore, the highest degradation efficacy of 90.4% was observed for 31BVBI nanocomposite than 13BVBI (81.4%), and 11BVBI (73.5%). Pristine BV and BI exhibited the least photocatalytic performance of 58.4%, and 68.4%, respectively. The photocatalytic performance of commercial TiO_2_-P25 powder was also evaluated in comparison to synthesized catalysts and was found to have 50.8%. The wide band gap of TiO_2_-P25, which renders it receptive to UV light only, may be the cause of its reduced performance. Hence, it is evident that the produced photocatalysts show higher photocatalytic performance than TiO_2_-P25 photocatalysts. Equation (5) was used to compute the rate constant for the observed catalytic performance.

ln(C/C_0_) = −kt (5)

Herein, C_0_, C, and k are the initial concentration of the pollutant, the concentration at time (t), and the rate constant, respectively. The reaction followed Langmuir-Hinshelwood kinetics model or pseudo-first-order kinetics as validated in Figure 5b because the lines were well fitted linearly with R^2^ ˃ 98.5%. The 31BVBI composite possesses the maximum rate constant of ~0.0159 min^−1^ owing to the formation of the heterojunction, while 11BVBI, 13BVBI, bare BV, and BI manifested the rate constant of 0.0081, 0.0094, 0.0053, and 0.0065 min^−1^, respectively. However, for TiO_2_-P25 powder, the rate constant was discovered to be 0.0042 min^−1^ which was the lowest of all the photocatalysts illustrating the superiority of the fabricated materials.

The synergy factor (R) was used to quantify the degree of a synergistic effect of the nanocomposites (Equation (6)).

(6) R=kBVkBIkBV+kBI

Here, k_BV_/k_BI_, k_BV_, and k_BI_ stand for the rate constant of coupled BV and BI, bare BV, and BI, respectively. R-value for the 11BVBI, 13BVBI, and 31BVBI nanocomposite was found to be 0.68, 0.79, and 1.34, respectively. Thus, it was gauged that 31BVBI had the highest R-value owing to the interfacial contact between BV and BI, and therefore higher photocatalytic performance.

#### 3.8.1. Impact of the Catalyst Amount

The decomposition efficacy is also dependent on the usage of the optimum amount of the photocatalyst for the degradation of the pollutant. Figure 5c depicts that the rate of deterioration of the pollutant initially increases from 67.2% to 90.4% as the catalyst concentration increases from 0.1 g/L to 0.5 g/L. This could be owing to the increment in the number of active centers on the surface of the catalyst. However, the reduction efficiency of the photocatalyst gets decreased by further increasing the catalyst amount to 0.6 g/L. The main factor responsible for decreasing the decomposition efficacy is the reduction in the ability of the photocatalyst to absorb light owing to the agglomeration of the particles with the augmentation in the catalyst amount. Additionally, the agglomerations may hinder the photons from penetrating the inner layers of the photocatalyst. As a result, the minimal number of particles are activated, which ultimately results in the generation of fewer holes (h^+^), hydroxyl (^•^OH), electrons (e^−^), and superoxide radicals (O_2_^−•^) [45]. Consequently, as the catalyst dose rises, the degradation rate tends to decrease. So, based on reduction efficiency, a dose of 0.5 g/L of the photocatalyst was selected as the optimal dosage for the subsequent studies. Hence, it can be concluded that even a small amount of 31BVBI is sufficient for the photocatalytic abatement of TC under exposure to sunlight.

#### 3.8.2. Influence of pH

The pH is a significant factor governing the degradation efficiency of the photocatalyst. The variation in pH affects the adsorption capacity, electric surface charge distribution, and ionization state of the photocatalyst [46]. The point zero charge (PZC) of the photocatalyst was validated to be 6.02 (Figure 5d). Therefore, at pH ˃ 6.02 and pH ˂ 6.02, the surface of the nanocomposite is negatively and positively charged, respectively. TC exists in the amphoteric state with 4 different pKa values (3.3, 7.7, 9.7, and 10.7). As a result, TC occurs as a positively charged molecule (TCH_3_^+^ and TCH_2_^+^) in pH < 3 owing to the protonation of the dimethyl ammonium group. It occurs as an anionic molecule (TC_2_^−^ and TCH^−^) at pH ˃ 7.7 due to deprotonation of diketone or tricarbon of phenol. While it exists in isoelectric form (TCH±) in pH between 3.3–7.7 owing to the subtraction of proton from the phenolic diketone group [47]. Due to repulsions developing between the anionic and cationic forms of TC and the negatively and positively charged surface of photocatalyst, respectively, the degradation efficiency of TC by 31BVBI photocatalyst is reduced in both acidic and alkaline environments. However, the zwitterionic form of TC and the negatively charged catalyst had an electrostatic affinity that improved the degradation ability of TC. Due to the affinity of TC for the surface of the photocatalyst, TC removal by the 31BVBI composite was therefore maximum at pH 7 (90.4%) and was greater in alkaline than in an acidic environment as depicted in Figure 5e.

#### 3.8.3. Effect of Different Light Sources

A comparable experiment was carried out in ideal conditions using UV light and visible light as the light sources. According to Figure 5f, TC deteriorated to 64.4, and 85.2% in UV, and visible light, respectively. However, the 31BVBI photocatalyst manifested the highest degradation rate of 90.4% when exposed to sunlight. As a result, the fabricated photocatalysts possess the ability to deteriorate refractory pollutants under the illumination of natural sunlight.

#### 3.8.4. Scavenger Experiment

**Figure 6:**
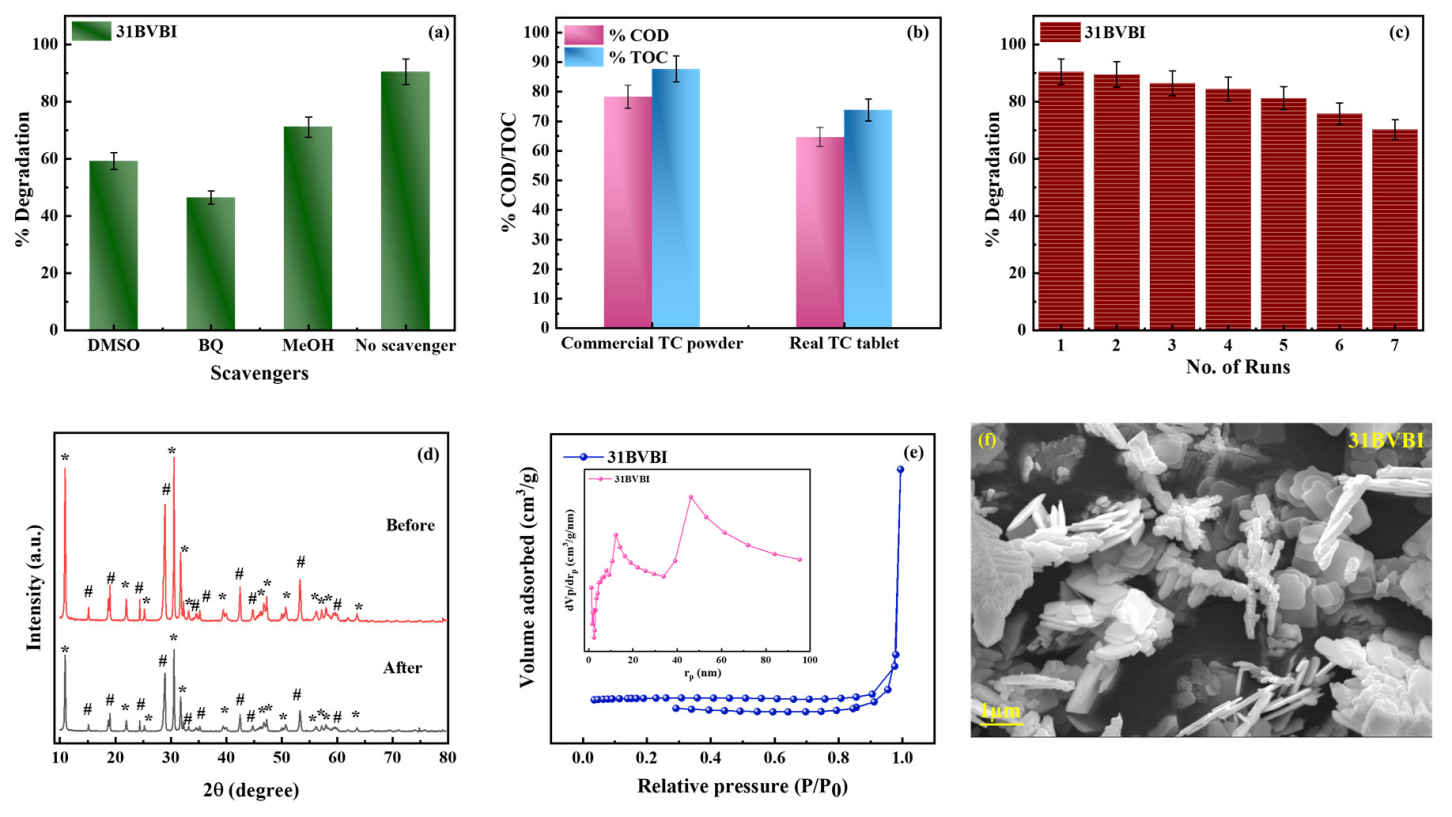
(**a**) Impact of different quenchers on the photocatalytic degradation of TC, (**b**)% COD/TOC analysis of commercial powder of TC, and real TC tablet, (**c**) reusability study, (**d**) XRD where # and * denote peaks of BV, and BI, respectively, (**e**) BET plot along with the inset having BJH graph, and (**f**) SEM image of used photocatalyst post degradation studies.

Photocatalytic degradation of TC involves a series of complex reactions driven by photogenerated radicals, or reactive oxygen species (ROS). Various species, mostly dissolved hydroxyl ions, oxygen, and water present in the solution capture photogenerated electrons and the holes to generate ROS i.e., h^+^, ^•^OH, O_2_^−•^, etc. [48]. Therefore, different scavengers e.g., methanol as h^+^, dimethyl sulfoxide (DMSO) as ^•^OH, and benzoquinone (BQ) as O_2_^−•^ scavenger were used to examine the role ROS responsible for the photocatalytic experiment. From Figure 6a, it can be inferred that the highest decomposition efficacy of 90.4% was observed for TC in the absence of any scavenging agent. The decomposition efficacy of TC by 31BVBI nanocomposite significantly are quenched from 90.4% to 46.2% in the presence of BQ. This indicates that the O_2_^−•^ is the major ROS responsible for the remediation of TC. DMSO had a comparable (60%), but less significant impact on the degradation process, indicating that ^•^OH plays a little but crucial function in the removal of antibiotics. These results specify that the O_2_^−•^ is the main active species for the removal of TC by the 31BVBI nanocomposite.

#### 3.8.5. Mineralization Study

Evaluation of the degree of mineralization of the organic matter along with the degradation of the organic pollutants is important. To determine the amount of mineralization of TC, TOC, and COD was carried out [49]. Elevated COD and TOC readings suggested that the antibiotic, TC contained a significant amount of organic matter. After carrying out photocatalytic treatment for 140 min under the irradiation of sunlight, % COD removal of 78.2 and 64.6% were observed for commercial TC solution and real TC tablet. Whereas, the % TOC removal was observed to be 87.7, and 73.8%, respectively (Figure 6b). The great effectiveness of the as-prepared photocatalyst can be validated by high COD and TOC removal efficacies. The successive organic intermediates that were formed during the process before the complete breakdown of TC to carbon dioxide and other simpler products caused the % TOC removal to be lower than % degradation [50]. Hence, the fabricated photocatalyst appears to be more effective in removing COD and TOC than the industrial physicochemical treatment.

#### 3.8.6. Reusability

Reusability and the optical stability of the photocatalyst are the key components for its practical use. The stability and the recyclability of the 31BVBI photocatalyst for the photocatalytic abatement of the TC were checked over 7 cycles and the results are demonstrated in Figure 6c. The photocatalyst was washed, filtered, and dried in the oven at 80 °C for 14 h before being used for the subsequent cycle. According to the results, the photocatalytic effectiveness of the 31BVBI photocatalyst has decreased roughly by 20.2%. This may be due to the loss of the photocatalyst during the washing and drying procedure, which could lower the surface catalytic property of the photocatalyst. In addition, aggregation of the nanocomposites may occur which could result in the reduced effective surface area and the number of reactive sites, thereby decreasing activity. Additionally, due to the occlusion of the pores and active sites of photocatalyst by TC and its intermediates after each cycle, the adsorptive surface activity of 31BVBI steadily diminishes [51]. However, the decrease in the photocatalytic activity was not significant even after 7 long runs. This validates that the photocatalyst is reusable and is potent for photocatalytic applications. XRD, SEM, and BET analyses were executed to determine the stability of 31BVBI photocatalyst after the degradation studies. Post the reusability studies, the XRD pattern of the nanocomposite displayed no discernible changes (Figure 6d). The peak positions and peak intensity of BI and BV were unaltered. No new diffraction peak corresponding to crystalline impurities was seen. Moreover, the BET physisorption analysis after the reusability studies validated that the photocatalyst had the mean pore diameter (17.26 nm), the specific surface area (57 m^2^/g), and mean pore volume (0.521 nm), respectively, as represented in Figure 6e. Even though the surface area is reduced but it was still sufficient to carry out photocatalytic decomposition. SEM executed after the recycling studies revealed that the morphology of the photocatalyst does not change (Figure 6f). Hence, the synthesized 31BVBI nanocomposite is stable, reusable, and can be utilized for practical applications. Table 2 tabularizes a comparative analysis of various photocatalysts for the photocatalytic remediation of TC in different light sources. On forming the heterojunction of BiVO_4_ and BiOBr, the recombination rate of the charge carriers of the photocatalyst is reduced. BiOBr has a unique lamellar structure with [Bi_2_O_2_]^2+^ sheets interspersed with two-layer bromine atoms (tetragonal matlockite structure). This shape offers enough room to polarize the associated kernel and orbital, making it easier to separate photo-produced carriers of the BiVO_4_ photocatalyst for effective photocatalytic/PEC activity. In the 3:1 BiVO_4_/BiOBr, the recombination rate is reduced as the band structures of both the photocatalysts are matched. Thereby, the band gap is lowered owing to which the visible light absorption capability is increased. Furthermore, 3:1 BiVO_4_/BiOBr has a higher surface area as the surface area is increased by increasing the amount of BiVO_4_ in the composite. Therefore, 3:1 BiVO_4_/BiOBr possesses the highest degradation activity. The highest decomposition efficacy was attained by the fabricated 31BVBI nanocomposite by employing a little amount of produced photocatalyst under the irradiation of natural sunlight and in the ideal reaction conditions in comparison to the current literature.

### 3.9. GC-MS Study

**Scheme 1:**
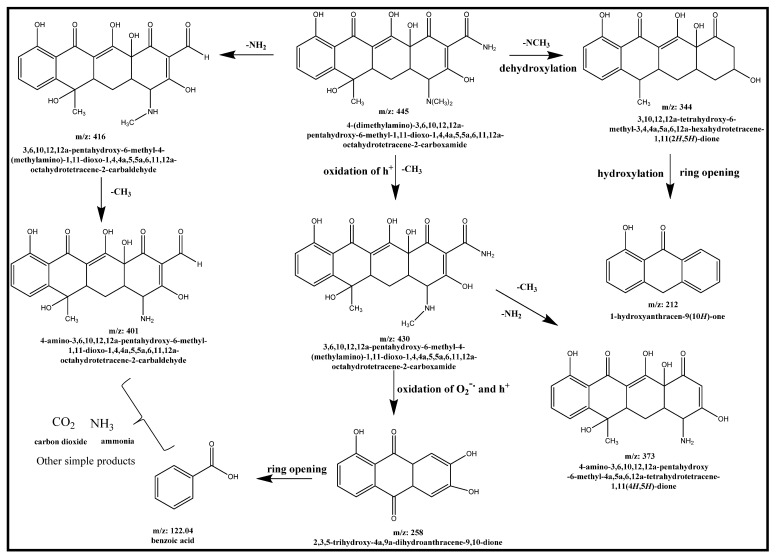
Proposed photocatalytic decomposition route of TC by 31BVBI nanocomposite.

A GC-MS analysis was performed to investigate the products and the intermediates formed during the photocatalytic remediation of TC by 31BVBI nanocomposite. Figure S3a,b illustrates the Gas chromatogram and mass spectrum, respectively. As represented in Scheme 1, the photocatalytic abatement process follows three degradation routes. In first degradation pathway, 3,6,10,12,12a-pentahydroxy-6-methyl-4-(methylamino)-1,11-dioxo-1,4,4a,5,5a,6,11,12a-octahydrotetracene-2-carbaldehyde was formed by the deamination of 4-(dimethylamino)-3,6,10,12,12a-pentahydroxy-6-methyl-1,11-dioxo-1,4,4a,5,5a,6,11,12a-octahydrotetracene-2-carboxamide. Further, 4-amino-3,6,10,12,12a-pentahydroxy-6-methyl-1,11-dioxo-1,4,4a,5,5a,6,11,12a-octahydrotetracene-2-carbaldehyde was formed by demethylation. Ring-opening reactions lead to the formation of CO_2_, NH_3_, and other simple products, thereafter [52]. In the second route dihydroxylation leads to the formation of 3,10,12,12a-tetrahydroxy-6-methyl-3,4,4a,5a,6,12a-hexahydrotetracene-1,11(2H,5H)-dione followed by the abstraction of NCH_3_ group from 4-(dimethylamino)-3,6,10,12,12a-pentahydroxy-6-methyl-1,11-dioxo-1,4,4a,5,5a,6,11,12a-octahydrotetracene-2-carboxamide. The 1-hydroxyanthracen-9(10H)-one was formed by the cleavage of ethyl, acetyl, hydroxyl group, and ring-opening reactions [53]. In the third decomposition route, formation of 3,6,10,12,12a-pentahydroxy-6-methyl-4-(methylamino)-1,11-dioxo-1,4,4a,5,5a,6,11,12a-octahydrotetracene-2-carboxamide occurred by the demethylation of 4-(dimethylamino)-3,6,10,12,12a-pentahydroxy-6-methyl-1,11-dioxo-1,4,4a,5,5a,6,11,12a-octahydrotetracene-2-carboxamide. Deduction of amide group and demethylation further lead to the formation of 4-amino-3,6,10,12,12a-pentahydroxy-6-methyl-4a,5a,6,12a-tetrahydrotetracene-1,11(4H,5H)-dione. Whereas, oxidation of h^+^ and O_2_^−•^ lead to the formation of 2,3,5-trihydroxy-4a,9a-dihydroanthracene-9,10-dion. The polarisation resulting from the intense adsorption of h^+^ to the electrons surrounding the benzene ring caused the stable benzene ring to break down and produce benzoic acid. Later it was degraded to give CO_2_, NH_3_, and other simple products [54].

**Table 2:** Comparison of different photocatalysts for the photocatalytic reduction of TC.

Photocatalyst	Contaminant’s Concentration (ppm)	Time of the Reaction (min)	Source of Light	Rate Constant k	Decomposition Efficiency (%)	Ref.
BiVO_4_/MoS_2_	TC (20 ppm)	120	Visible Light	-	68.83	[55]
BiVO_4_/Fe_3_O_4_/CdS	TC (10 ppm)	90	Visible Light	0.0232 min^−1^	87.37	[56]
BiVO_4_/Cu/g-C_3_N_4_	TC	120	Visible Light	0.0110 min^−1^	69.00	[57]
BiOBr/carbon fibre/g-C_3_N_4_	TC (10 ppm)	120	Visible Light	0.0150 min^−1^	86.10	[58]
CuInS_2_/Bi_2_MoO_6_	TC (10 ppm)	120	Visible Light	0.0150 min^−1^	84.70	[59]
MnTiO_3_/Ag/g-C_3_N_4_	TC	120	Visible Light	-	61.00	[60]
31BVBI	TC (10 ppm)	140	Sunlight	0.0159 min^−1^	90.40	current work

### 3.10. PEC Measurements

**Figure 7:**
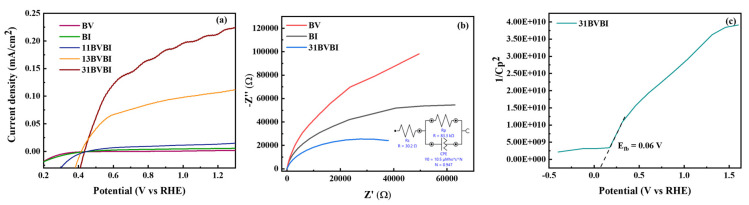
(**a**) LSV of 15c-BV, BI, 11BVBI, 13BVBI, and 31BVBI in solar light, (**b**) Nyquist study of BV, BI, and 31BVBI in light condition, and (**c**) Mott-Schottky graph of 31BVBI composite.

LSV analyses with 5, 10, and 15 layer coating (5c, 10c, and 15c) were conducted to assess the performance of the fabricated photocatalysts towards PEC hydrogen generation and to investigate the photo-response as presented in Figure S3a–e. Due to its higher photocurrent density of 0.2198 mA/cm^2^ compared to other photocatalysts, the 15c was designed to be the best among the others. 15c-11BVBI, 13BVBI, and 31BVBI unveiled greater photocurrent densities of 0.0139, 0.1130, and 0.2198 mA/cm^2^, respectively at 1.23 V vs. RHE than bare BV (0.0015 mA/cm^2^) and BI (0.0052 mA/cm^2^). Figure 7a displays the LSV spectra of the fabricated 15c photocatalysts in light. The results imply that among all the composites, the 15c-31BVBI composition had the maximum photocurrent density.

To scrutinize the kinetics of the interfacial photogenerated charge migration of the fabricated photocatalysts in both light and dark at the electrode/electrolyte interface, EIS was performed and the results are illustrated in Figure S4a–e [60]. Nyquist plots reveal that the controlled BV and BI electrode has a significantly greater charge transfer resistance (R_ct_) than the 31BVBI electrode with the optimal coating as represented in Figure 7b. Results are in agreement with LSV observation [61]. For all of the prepared photoelectrodes, a decrease in R_ct_ value was detected in the light compared to the dark. The R_ct_ value of the 31BVBI electrode was found to be 83.3 kΩ, which otherwise was 388 and 294 kΩ for BV and BI, respectively under the solar light.

**Figure 8:**
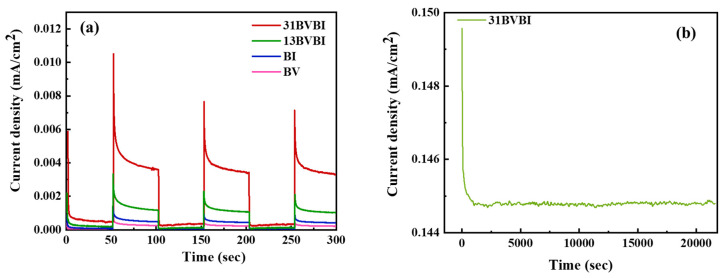
(**a**) Transient current density graph of the fabricated BV, BI, 13BVBI, and 31BVBI electrodes at 1.23 V vs. RHE with 50 s time intervals, and (**b**) Stability examination of 31BVBI electrode for 21,600 s.

The flat band potential (E_fb_) of the 31BVBI electrode was further calculated using the Mott–Schottky measurements to comprehend the charge transfer mechanism. It was observed that 31BVBI had a positive slope, indicating that the photocatalyst exhibit n-type semiconductor properties and behaves as a photoanode [62]. The x-intercept of the Mott–Schottky plot can be used to estimate the flat band potential and it was validated to be 0.06 V as shown in Figure 7c. Chopped LSV experiments under 50 s pulse time in both solar light and dark were carried out to investigate the photo-response behavior of the fabricated BV, BI, and 31BVBI photocatalyst at 1.23 V vs. RHE (Figure 8a). The produced electrodes manifest an instant increase in current in the light to a steady state, followed by a return to the ground when the light was switched off [63]. Their photocurrent values were consistently steady during all light-switching processes, demonstrating that they are consistently stable when exposed to light. The 31BVBI exhibited a higher current density of 0.011 mA/cm^2^, therefore, confirming high charge separation and transport efficiency over BV (0.00049 mA/cm^2^), and BI (0.0011 mA/cm^2^).

Chronoamperometry measurements were carried out for 21,600 s (6 h) to assess the stability of fabricated 31BVBI electrode. As illustrated in Figure 8b, after 6 h, photocurrent was seen to be almost steady, and just a slight drop was observed indicating that the fabricated electrode was stable. The amount of H_2_ evolved was also measured theoretically from Equation (7) during 6 h stability study of the 31BVBI electrode and found to be 1.864 mmolcm^−2^ s^−1^ at 1.23 V versus RHE. 

Number of moles of Hydrogen produced =

(7) $$\frac{Faradic\;efficiency*\;Charge}{2*Faeadic\;constant}$$

Thus, the fabricated electrode is stable enough for a longer period to evolve the hydrogen via PEC water splitting. Table 3 summarizes the PEC activity of different photocatalysts in comparison to the 31BVBI photocatalyst.

### 3.11. Photo(electro)catalytic Mechanism

**Scheme 2:**
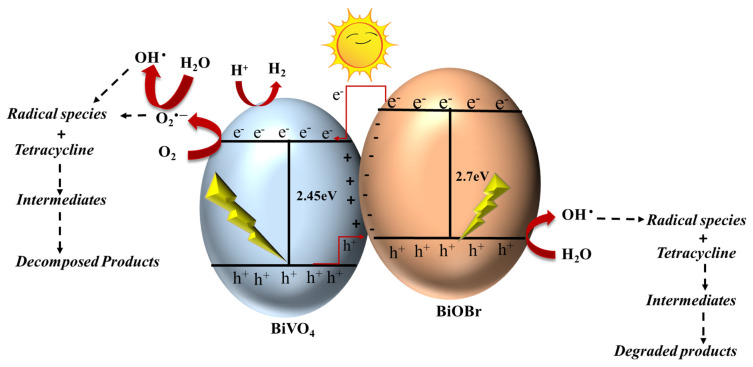
Proposed photo(electro)catalytic route for the reduction of pollutants, and the evolution of hydrogen by water splitting.

The photogenerated charge transfer mechanism for a photo(electro)catalytic activity of the fabricated photocatalyst is provided in Scheme 2. The Fermi level of BV (n-type semiconductor) is positioned slightly below its conduction band while, the Fermi level of BI (p-type semiconductor) is just above its valence band. On the formation of heterojunction, the Fermi levels of BV and BI will gradually migrate closer to one another, eventually attaining the same potential value. BV has a greater negative Fermi level than BI resulting in the movement of the Fermi level of BV to a positive and BI to a negative direction until equilibrium is obtained. The conduction and valence band potential of BV will change positively in response to the positive migration of its Fermi level. These bands will therefore incline upward. At the interface of BV and BI electric field will be created directing from BV to BI. The electrons existing in the valence band edge potential of BV and BI get excited to their respective conduction bands when illuminated by solar light. The occurrence of the electric field at the interface of the semiconductors will cause the electrons to migrate to the conduction band of BV from that of BI. Thus, the photogenerated holes present in the valence band edge of BI form a hole-rich layer, while the electrons in the conduction band edge of BV form an electron-rich layer. Heterojunction formation can significantly increase the charge separation efficiency, thereby promoting photocatalytic TC degradation and PEC hydrogen production by effectively inhibiting the secondary recombination of the charges. For the degradation of TC, electrons present in the conduction band of BV can interact with O_2_ to form O_2_^−•^ and later react with H_2_O to produce OH^•^. This OH^•^ radical can directly oxidize and reduce the organic contaminant. Additionally, the h^+^ present in the valence band of BI can combine with water to generate OH^•^, which can further oxidize harmful pollutants into less dangerous byproducts. For the PEC water splitting, H^+^ ions can be reduced to form H_2_ at the counter electrode.

## 4. Conclusions

In this study, BiVO_4_/BiOBr heterojunction photocatalysts with various mole ratios were successfully fabricated using the hydrothermal process. Characterization studies revealed that the hetero composite exhibited a lower rate of charge recombination, a large surface area, and a narrow band gap, which are essential for effective photocatalysis and photoelectrocatalysis. The impact of different mole ratios on the photocatalytic degradation of the recalcitrant antibiotic, TC, and PEC water splitting for hydrogen evolution under solar light was thoroughly investigated. Various aspects such as photocatalyst concentration, pH effects, kinetic studies, light source effects, scavenger studies, reusability, LSV, Mott-Schottky analysis, EIS, and chronoamperometry were all examined. Among the synthesized photocatalysts, the 31BVBI composition exhibited the highest photocatalytic TC degradation efficiency of 90.40% (rate constant 0.0159 min^−1^) under the illumination of sunlight for 140 min. Also, it exhibited the highest photocurrent density of 0.2198 mA/cm^2^ at 1.23 V vs. RHE for the photoelectrocatalytic water splitting experiments under solar light irradiation. To demonstrate the superiority of the fabricated nanocomposites, their performance was compared to that of commercial TiO_2_-P25. The primary quenching species responsible for the decomposition process were found to be O_2_^−•^ radicals. The photocatalysts showed excellent reusability, with the ability to be easily recovered and reused for up to seven cycles. Post-degradation investigations using BET, SEM, and XRD analyses revealed that the surface area properties, morphology, and crystallinity of the nanocomposites remained unchanged. The GC-MS analysis confirmed the formation of products and intermediates during the decomposition of TC. The high values of COD and TOC removal indicated the effective mineralization of TC by the prepared composite, highlighting its potential for removing harmful antibiotics. Stability studies further confirmed the electrode’s durability for a prolonged period of 21,600 s.

In conclusion, the BiVO_4_/BiOBr photocatalyst, particularly the 31BVBI composition, exhibits significant potential for degrading harmful contaminants and facilitating PEC water splitting, offering promising applications in the field of water purification and hydrogen generation.

## Figures and Tables

**Table 1:** The surface area characteristics of fabricated catalysts.

Sample	Specific Surface Area (m^2^/g)	Mean Pore Volume (cm^3^/g)	Pore Diameter (nm)
BV	32	0.551	13.62
BI	28	0.520	12.72
11BVBI	50	0.572	16.37
13BVBI	54	0.661	19.14
31BVBI	63	0.686	21.46

**Table 3:** Comparison of PEC activity of 31BVBI with the reported literature.

S.No.	Photocatalysts	Electrolyte	Electrode Type	Photocurrent Density	Moles of H_2_ Produced	References
1.	BiOBr nanosheet arrays	0.1 M Na_2_SO_4_	Photoanode	0.0069 mA/cm^2^ at 0.9 V vs. RHE	-	[64]
2.	Bi_2_S_3_/BiOBr/TiO_2_	0.5 M Na_2_SO_4_	Photoanode	0.0091 mA/cm^2^ at 1.2 V vs. RHE	0.0047 µmolcm^−2^ s^−1^	[65]
3.	BiVO_4_ Nanowire	0.1 M potassium phosphate buffer	Photoanode	0.0093 μA/cm^2^ at 1.2 V vs. RHE	-	[66]
4.	g-C_3_N_4_/BiVO_4_	Lake wastewater	Photoanode	9.6800 mA/cm^2^ at 1.0 V vs. Ag/AgCl	0.0059 µmolcm^−2^ s^−1^	[67]
5.	BiVO_4_/BiOBr	0.5 M Na_2_SO_4_	Photoanode	0.2198 mA/cm^2^ at 1.23 V vs. RHE	1.8640 mmolcm^−2^ s^−1^	Current Works

## Data Availability

The datasets generated during the current study are available from the corresponding authors upon reasonable request.

## Supplementary Materials

The following supporting information can be downloaded at: https://www.mdpi.com/article/10.3390/ma16165661/s1, Supplementary information embraces the photoluminescence spectra, characterization methods, XRD spectrum of 11BVBI, and 13BVBI nanocomposite, gas chromatogram, the mass spectrum of the decomposed products of TC by 31BVBI nanocomposite, LSV graphs of the different coatings of synthesized photocatalysts, Nyquist plots of different photocatalysts with 15c coating in both light and dark.
