# Supplementary material for: Revolutionizing the Role of Solar Light Responsive BiVO4/BiOBr Heterojunction Photocatalyst for the Photocatalytic Deterioration of Tetracycline and Photoelectrocatalytic Water Splitting
Source: Materials (Basel). 2023 Aug 17;16(16):5661. doi: 10.3390/ma16165661 (PMC10456310; doi:10.3390/ma16165661)
Supplement: Supplementary file 1 [file materials-16-05661-s001.zip › materials-2552043-supplementary.pdf]

Supporting Materials

Revolutionizing the Role of Solar Light Responsive $BiVO_4$/BiOBr Heterojunction Photocatalyst for the Photocatalytic Deterioration of Tetracycline and Photoelectrocatalytic Water Splitting

Shelly Singla [1,2], Pooja Devi [1,*] and Soumen Basu [2,*]

[1] Materials Science and Sensor Application, Central Scientific Instruments Organisation, Chandigarh 160030, India; shellysingla96@gmail.com
[2] School of Chemistry and Biochemistry, Thapar Institute of Engineering and Technology, Patiala 147004, India
* Correspondence: poojaiitr@csio.res.in (P.D.); soumen.basu@thapar.edu (S.B.)

S1.1. Characterization methods

PAN analytical X′ Pert-Pro X-ray diffractometer with the radiation of Cu Kα, operated at 45 kV with a scan range of 10-80°, step size 0.026°, and wavelength = 1.5406 was used to assess the materials' X-ray diffraction analysis (XRD). The nitrogen sorption analysis was performed using a Microtrac Belsorp Mini-II (Bel, Japan, Inc) surface area analyzer. Prior to the evaluation, the samples were pre-treated in a nitrogen atmosphere at 100°C for 5 h to prevent the trapping of undesirable pollutants and gases. The Brunner-Emmet-Teller (BET) method and Barrett Joyner-(BJH) Halenda's approach was used to inspect the pore size distribution and surface area curves, respectively. The pH was measured with a pH meter from Eutech (Singapore), model cyber scan pH 1100. The kinetics of samples by photocatalytic degradation of organic pollutants under sunshine by Analytik Jena spectrophotometer. UV–Vis diffuse reflectance spectroscopy (DRS) were examined using a Shimadzu UV 2600 spectrophotometer in diffused absorbance mode. At the excitation wavelength of 350 nm of the as-prepared catalyst, photoluminescence (PL)

spectroscopic experiments were performed using the Perkin Elmer LS-55, USA PL spectrometer. The oxidation states of metal oxides were determined using the PHI5200 X-ray photoelectron spectroscopy (XPS) system, which included an Omicron ESCA apparatus and a monochromatic Al K X-ray source (1486.7eV). JEOL instrument JSM-6510 was employed with the voltage of 15 kV to assess the morphological studies of as-prepared catalyst along with color mapping images. SEM-EDS was used to inspect the color spectra of the composite. TOC was measured using a TOC analyzer (Model: Multi N/C 2100 BU, Analytik Jena AG Corporation).

The degraded products obtained after photocatalytic reduction of the hazardous pollutant, TC were examined using GC-MS. The aqueous mixture was collected following the partition of the photocatalyst and decomposition reaction. Dichloromethane (DCM) was used to dissolve the residue and the former was then evaporated during the drying at 35°C. Shimadzu QP-2010 plus ultra-instrument with split/split less injector was utilized for analysis utilizing the optimized method: ion source temperature = 220°C, split mode injector with injector temperature = 260°C, sample injection volume = 1 μL, and helium carrier gas (flow rate = 1.23 mL/min). Oven temperature was kept at 100°C for a 2 min before being augmented to 300°C for an additional 18 min. Mass spectra was monitored between a range m/z = 40–650.

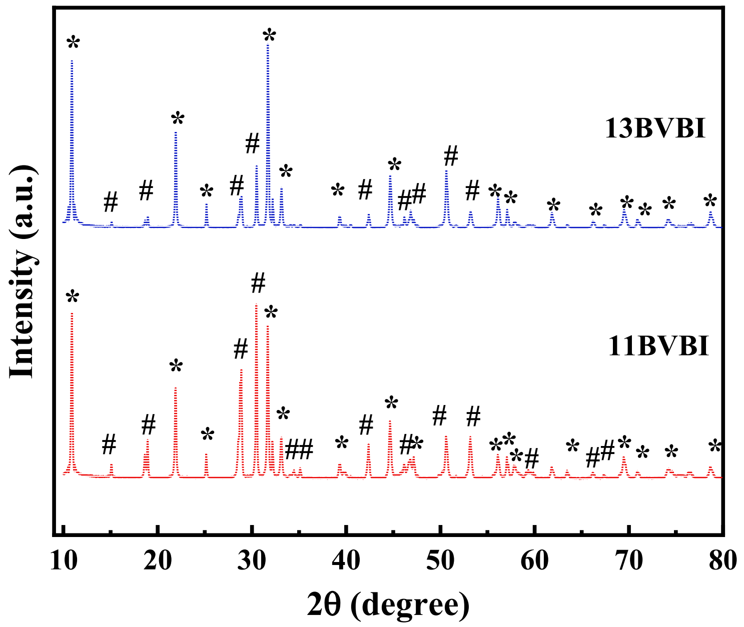

Figure S1 XRD spectra of (a) 11BVBI, and (b) 13BVBI nanocomposite. # and * denote peaks of BV, and BI, respectively.

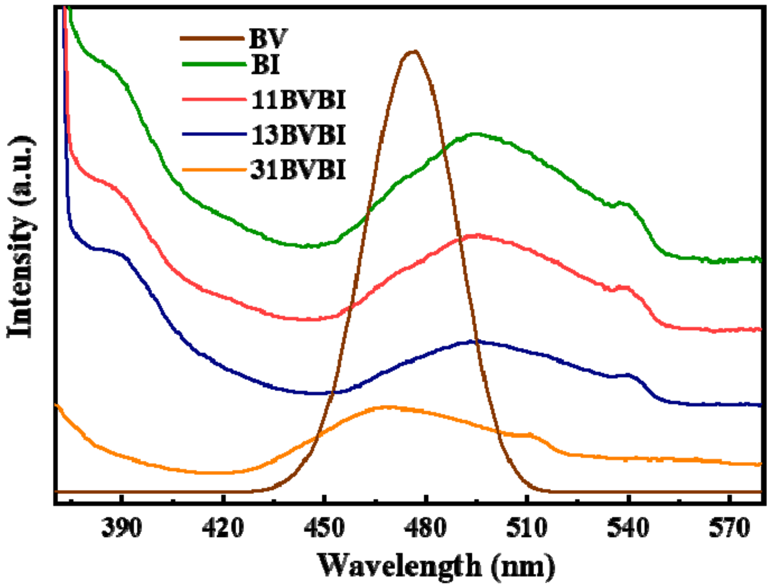

Figure S2 PL spectra of fabricated photocatalysts.

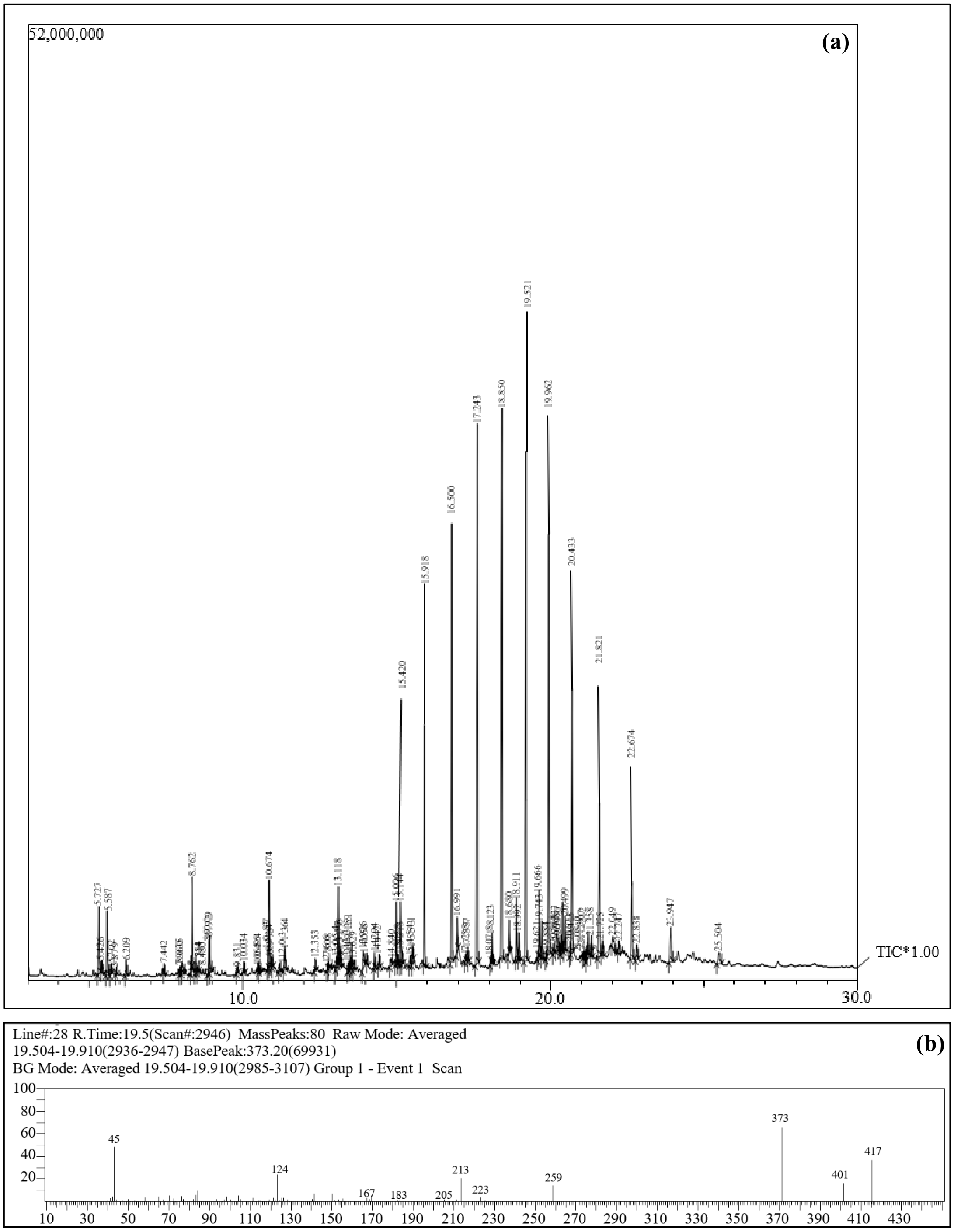

Figure S3(a) Gas chromatogram, (b) Mass spectra of the degraded products formed after the photocatalytic degradation of TC by 31BVBI photocatalyst.

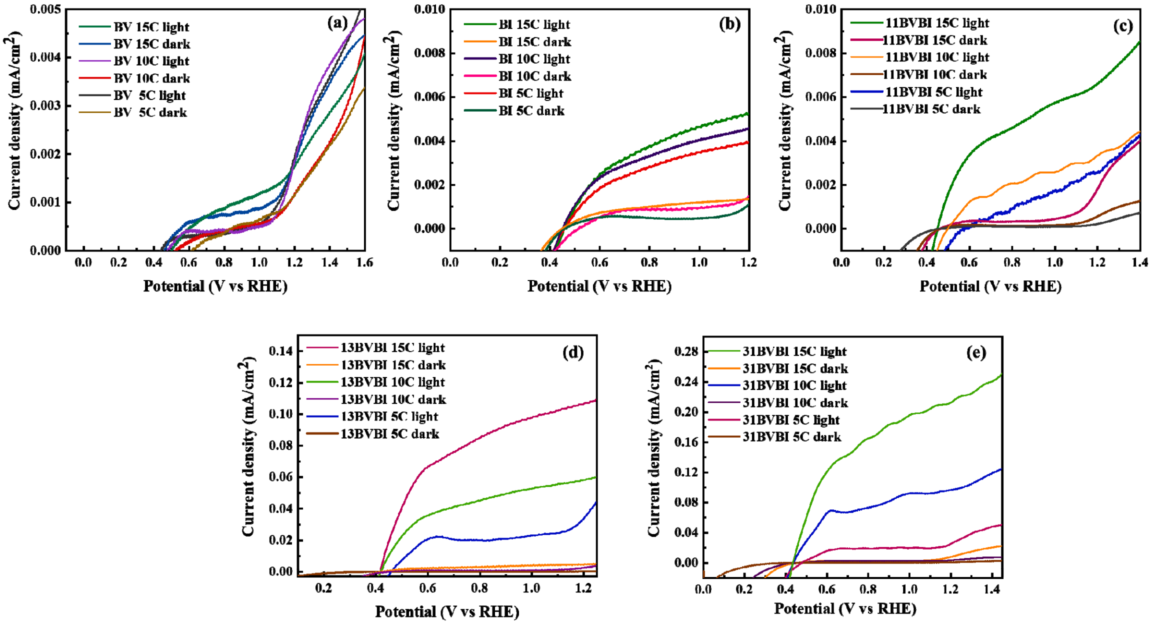

Figure S4 LSV of different coating layers of (a) BV, (b) BI, (c) 11BVBI, (d) 13BVBI, and (e) 31BVBI in dark and light condition.

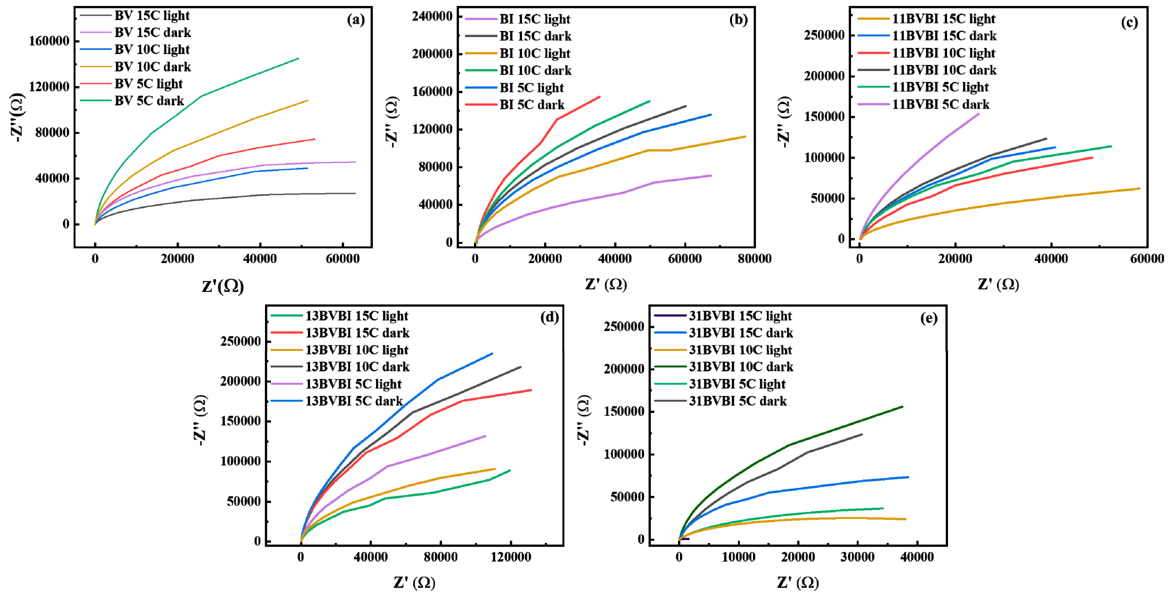

Figure S5 Nyquist plot of 15 coatings of (a) BV, (b) BI, (c) 11BVBI, (d) 13BVBI, and (e) 31BVBI in dark and light condition.
